# Robust Network Inhibition and Decay of Early-Phase LTP in the Hippocampal CA1 Subfield of the Amazon Rodent *Proechimys*

**DOI:** 10.3389/fncir.2018.00081

**Published:** 2018-10-04

**Authors:** Selvin Z. Reyes-Garcia, Antônio-Carlos Guimarães de Almeida, Nancy N. Ortiz-Villatoro, Fulvio A. Scorza, Esper A. Cavalheiro, Carla A. Scorza

**Affiliations:** ^1^Disciplina de Neurociência, Departamento de Neurologia e Neurocirurgia, Escola Paulista de Medicina, Universidade Federal de São Paulo, São Paulo, Brazil; ^2^Departamento de Ciencias Morfológicas, Facultad de Ciencias Médicas, Universidad Nacional Autónoma de Honduras, Tegucigalpa, Honduras; ^3^Laboratório de Neurociência Experimental e Computacional, Departamento de Engenharia de Biossistemas, Universidade Federal de São João del-Rei, São João del-Rei, Brazil

**Keywords:** synaptic plasticity, long-term potentiation, CA1 region, hippocampal, GABA modulators, neural inhibition

## Abstract

**Background**: Diverse forms of long-term potentiation (LTP) have been described, but one of the most investigated is encountered in the glutamatergic synapses of the hippocampal *cornu Ammonis* (CA1) subfield. However, little is known about synaptic plasticity in wildlife populations. Laboratory animals are extremely inbred populations that have been disconnected from their natural environment and so their essential ecological aspects are entirely absent. *Proechimys* are small rodents from Brazil’s Amazon rainforest and their nervous systems have evolved to carry out specific tasks of their unique ecological environment. It has also been shown that long-term memory duration did not persist for 24-h in *Proechimys*, in contrast to Wistar rats, when both animal species were assessed by the plus-maze discrimination avoidance task and object recognition test.

**Methods**: In this work, different protocols, such as theta burst, single tetanic burst or multiple trains of high frequency stimulation (HFS), were used to induce LTP in hippocampal brain slices of *Proechimys* and Wistar rats.

**Results**: A protocol-independent fast decay of early-phase LTP at glutamatergic synapses of the CA1 subfield was encountered in *Proechimys*. Long-term depression (LTD) and baseline paired-pulse facilitation (PPF) were investigated but no differences were found between animal species. Input/output (I/O) relationships suggested lower excitability in *Proechimys* in comparison to Wistar rats. Bath application of d-(-)-2-amino-5-phosphonopentanoicacid (D-AP5) and CNQX prevented the induction of LTP in both *Proechimys* and Wistar. However, in marked contrast to Wistar rats, LTP induction was not facilitated by the GABA_A_ antagonist in the Amazon rodents, even higher concentrations failed to facilitate LTP in *Proechimys*. Next, the effects of GABA_A_ inhibition on spontaneous activity as well as evoked field potentials (FPs) were evaluated in CA1 pyramidal cells. Likewise, much lower activity was detected in *Proechimys* brain slices in comparison to those of the Wistar rats.

**Conclusions**: These findings suggest a possible high inhibitory tone in the CA1 network mediated by GABA_A_ receptors in the Amazon rodents. Currently, neuroscience research still seeks to reveal molecular pathways that control learning and memory processes, *Proechimys* may prove useful in identifying such mechanisms in complement to traditional animal models.

## Introduction

Synaptic transmission is the hub of neural communication. Essential for several brain functions, synaptic plasticity, a key attribute of synaptic transmission, is a dynamic modification in the efficacy of synaptic strength, converting information processing into neural circuits (Vitureira and Goda, 2013). By controlling the synaptic strength, glutamatergic and GABAergic neurotransmission systems are very plastic and ensure brain homeostatic regulation. In this context, it has remained an open question how one does conciliate the properties of a particular and accurately wired brain with the competence to acquire knowledge, learning and memory. The pioneer in introducing the concept of synapse specificity was Ramón y Cajal (1894) who proposed that learning would be the result of the modifications in the connection strength. The concept was later refined by Hebb (1949) and is frequently shortened to “neurons wire together if they fire together” (Löwel and Singer, 1992). The discovery of the phenomenon of long-lasting potentiation by Bliss and Lømo (1973) was the first description of synaptic plasticity. Since then, long-lasting types of synaptic plasticity discriminated as Hebbian plasticity (when presynaptic activity is strictly followed by postsynaptic activity), comprising, among others, long-term potentiation (LTP), have been largely investigated as attractive cellular mechanisms underlying some forms of learning and memory (Bliss and Lømo, 1973; Ito and Kano, 1982; Bliss and Collingridge, 1993; Collingridge et al., 2010; Bliss and Cooke, 2011; Lüscher and Malenka, 2012; Mayford et al., 2012). Diverse forms of LTP have been described, but one of the most investigated is encountered in the glutamatergic synapses of the hippocampal *cornu Ammonis* (CA1) subfield. However, little is known about synaptic plasticity in wildlife populations (Amrein, 2015). Laboratory animals are extremely inbred populations that have been disconnected from their natural environment and so their essential ecological aspects are entirely absent (Klaus and Amrein, 2012; Keifer and Summers, 2016). Comparative studies may add to our comprehension of the basic functions of the brain so the use of non-standard populations can be instructive in addition to traditional animal models (Keifer and Summers, 2016).

*Proechimys* are small rodents from Brazil’s Amazon rainforest and their nervous systems have evolved to carry out specific tasks of their unique ecological environment (Carlson, 2012; Keifer and Summers, 2016). Previous work of our group has showed that these wild rodents display a fast decaying LTP and poor long-lasting memory process (Guimarães Marques et al., 2018). It has also been shown that long-term memory duration did not persist for 24-h in *Proechimys*, in contrast to Wistar rats, when both animal species were assessed by the plus-maze discrimination avoidance task and object recognition test (Guimarães Marques et al., 2018). In this line, studies have suggested a link between the maintenance of LTP and long-term memory maintenance (Pastalkova et al., 2006; Sacktor, 2008). The decay of LTP is still a poorly understood process but dynamic network inhibition has been implicated in the loss of synaptic potentiation over time (Dong et al., 2015). In the present work, using different standard protocols for inducing LTP, we confirmed the protocol-independent rapid decay of the early-phase LTP in *Proechimys*’s glutamatergic synapses of the CA1 hippocampal subfield. Furthermore, our findings from the use of modulators of GABAergic and glutamatergic transmission suggested a high inhibitory tone in this CA1 network mediated by GABA_A_ receptors in the wild Amazon rodents, which could play a role in the fast decay of LTP encountered in this study.

## Materials and Methods

### Animals

Male rodents derived from wild *Proechimys* trapped at the Brazilian Amazon forest were bred in a colony established at the Neuroscience Laboratory’s facility (Federal Technical Register-IBAMA number 1561643) of Escola Paulista de Medicina/Universidade Federal de São Paulo (EPM/UNIFESP). Male Wistar rats were bred in the biotery of the University EPM/UNIFESP (CEDEME). All adult animals used in the experiments, weighing 250–300 g, were housed under environmentally controlled conditions (22°C ± 1°C), 12/12 h light-dark cycle (lights on at 7 a.m), with water and food *ad libitum*. All animal procedures were carried out in accordance with the ethical and practical principles of the use of laboratory animals with the approval of the ethical committee of EPM/UNIFESP (CEUA 9108060315). Precautions were taken to minimize the number of animals used in the experiments.

### Brain Slice Preparation

Brain slices were prepared as described by Guimarães Marques et al. (2018). Briefly, 1% isoflurane in 70% N_2_O and 30% O_2_ was used to anesthetize animals. Next, adult Wistar and *Proechimys* rodents were decapitated and brains immediately extracted. Brain were sliced in ice-cold artificial cerebrospinal fluid (aCSF) at 4°C ± 0.5°C temperature composed by (in mM) NaCl 129, NaHCO_3_ 21, KCl 3, CaCl_2_ 1.6, MgSO_4_ 1.8, NaH_2_PO_4_ 1.25 and glucose 10, saturated with 95% O_2_ and 5% CO_2_. Horizontal hippocampal slices were cut at 400 μm thick using a vibratome (LEICA VT 1200S). Slices were immediately transferred to an interface chamber perfused with aCSF at 36°C ± 0.5°C (flow rate: 1.5–2.0 ml/min, pH 7.4, osmolarity: 295–300 mOsmol/L) and recordings started 2–3 h after slice recovering.

### *In vitro* Electrophysiology—Stimulation and Recording

Extracellular field potentials (FPs) were obtained from the *stratum radiatum* (field excitatory postsynaptic potentials, fEPSPs) and *pyramidale* population spike (PS) of CA1 subfield. Using a stimulating bipolar twisted electrode (Ni-Cr wire with 50 μm diameter tip) positioned in the *stratum radiatum* of the CA1 hippocampal region, orthodromic stimuli were delivered in the Schaffer collateral axons. The recording electrode was made by a chlorinated silver wire placed into a glass capillar filled with 154 mM NaCl (5–10 MΩ). Low-pass filtered (3 kHz) data were digitized at 10 KHz then stored on computer disk through a CED 1401 interface for off-line analysis utilizing Spike 2 v6.09 (CED-1401, Cambridge, UK). Prior to the LTP/long-term depression (LTD) induction, input/output (I/O) relationships of stimulus intensity against to fEPSPs magnitude were carried out to obtain the maximal amplitude subsequently to uniform rises of stimulus intensity up to no further augmentation of the fEPSP amplitude. Stimulus intensities were adjusted to elicit a fEPSP slope of 50%–60% of the maximum accessed from the I/O. I-O curves for fEPSP slope, fiber volley (FV) amplitude and PS amplitude were recorded before and after the induction of LTP by four high frequency stimulation (4HFS). Field EPSP slope was measured after the FV with the intention of avoiding the impact of other sources of current flow. Paired pulse facilitation (PPF) was assessed at interstimulus intervals (ISIs) of 20, 40, 80, 160 and 320 ms in both groups of animals. To PPF, 10 trials at each interval were assessed. PPF (%) was determined as the ratio between the second pulse-evoked of fEPSP and the first one. LTP was defined as alterations in fEPSP slope higher than 20%. The high frequency stimulus was applied after 20 min of stable baseline recording. LTP was induced by: (1) a single train of HFS, 100 Hz in one second; (2) by four trains of HFS with 20 s inter-train interval; and (3) theta burst stimulation (TBS) protocol consisted of 10 bursts repeated at 200 ms intervals, with four pulses at 100 Hz for each burst. All protocols were delivered to the Schaffer collaterals and responses were recorded for 80 min. LTP induction was assessed in different set of experiments by observing modifications in fEPSP after adding the NMDA receptor blocker d-(-)-2-amino-5-phosphonopentanoicacid (D-AP5; 100 μM; Tocris, Bristol, UK), CNQX disodium salt (10 μM; Tocris, Bristol, UK) or the GABA_A_ receptor blockers bicuculline methiodide (10 μM, 30 μM, 60 μM; Sigma-Aldrich, St. Louis, MO, USA) and picrotoxin (0.1 mM, Research Biochemicals International, Natick, MA, USA) or the GABA_A_ receptor positive allosteric modulator diazepam (1 μM, Compaz^®^, Cristália, Brazil), in the perfusion bath, 10 min before until 20 min after LTP induction. In the experiments with bicuculline and picrotoxin, the concentrations of Ca^2+^ and Mg^2+^ were increased to 4 mM to minimize polysynaptic and burst discharges (Chapman et al., 1998). In the experiments with CNQX, magnesium-free aCSF were used (Muller et al., 1988). Additionally, spontaneous as well as evoked field activity were both investigated through the effect of GABA_A_ antagonism on brain slices using bicuculline (10 μM, 30 μM and 60 μM).

### Statistical Analysis

Data were analyzed using custom-made scripts (^©^Jan-Oliver Hollnagel, MATLAB R2013b; Salar et al., 2016). The fEPSP slope was measured between 20% and 80% of its maximal amplitude, the amplitude of the PS was examined by measuring the difference between the negative peak and the middle point located between the line connecting the first and second positive peaks. In order to verify the potentiation after HFS, fEPSP slopes were normalized relative to the averaged baseline response. For statistical analysis, all data were reported as mean ± standard error of the mean (SEM). Statistical significance was assessed by non-parametric Wilcoxon Signed Rank test or Mann–Whitney U test. For PPF Friedman’s two-way analysis of variance with repeated measures was used. Sets of electrophysiological experiments with three groups were analyzed using Kruskal-Wallis test, followed by Dunn’s *post hoc* multiple comparisons test, *p* < 0.05 (*) was considered to indicate a significant difference.

## Results

### Hippocampal Synaptic Plasticity

**Figure 1:**
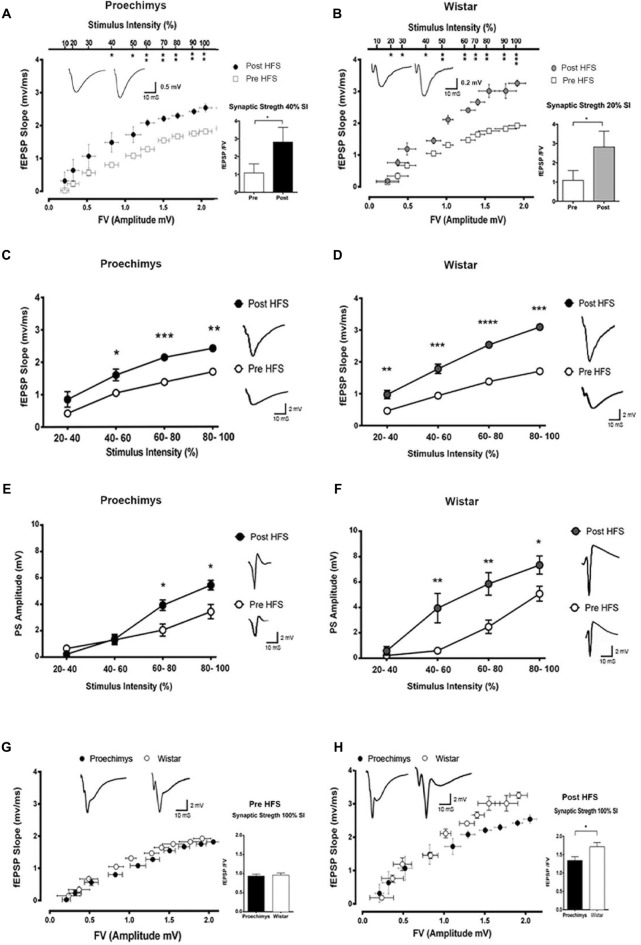
Input/output (I/O) relationships in *Proechimys* and Wistar rodents before and after four high frequency stimulation (4HFS). **(A,B)** Analysis of fiber volley (FV) amplitude vs. field excitatorypostsynaptic potential (fEPSP) slope at each level of stimulus intensity (% of the maximum). Insets, average and recordings of synaptic strength connection (fEPSP/FV amplitude). **(A)** In *Proechimys*, potentiation started at 40% of maximal stimulus intensity (MSI; *W* = 16, *p = 0.0469). **(B)** In Wistar, at 20% of MSI, (*W* = 21, *p = 0.0156). **(C,D)** Stimulus intensity vs. fEPSP slope. **(C)** In *Proechimys*, 40%–100% of MSI resulted in enhancement of fEPSP slope after 4HFS; for example, for 40%–60% of MSI: 1.048 ± 0.09 mV/ms pre-4HFS and 1.604 ± 0.18 mV/ms post-4HFS (*W* = 34, *p = 0.0078). **(D)** In Wistar, 20%–100% of MSI resulted in enhancement of fEPSP slope after 4HFS, example at 20%–40% of MSI: 0.47 ± 0.09 mV/ms pre-4HFS and 0.98 ± 0.13 mV/ms post-4HFS (*W* = 50, **p = 0.0237). Insets in **(C,D)** are examples of fEPSP recorded at 40%–60% of stimulus intensity pre and post 4HFS. **(E,F)** Stimulus intensity vs. population spike (PS) amplitude. **(E)** In *Proechimys*, enhancement in pyramidal cell firing was found at 60%–100% of MSI, example of amplitude at 60%–80% of MSI: 2.05 ± 0.46 mV pre-4HFS and 3.93 ± 0.39 mV post-4HFS, Wilcoxon test (*W* = 58, *p = 0.0105). **(F)** In Wistar, differences were found at 40%–100% of MSI, example at 40%–60% of MSI: 0.59 ± 0.20 mV pre-4HFS and 3.94 ± 1.16 mV post-4HFS, Wilcoxon test (*W* = 51, **p = 0.0059). Insets are example or PS recordings at 60%–80% of MSI. **(G,H)** Comparison of synaptic strength between both animal species. **(G)** No differences were encountered between *Proechimys* and Wistar before tetanic stimulation. **(H)** After 4HFS, higher enhancement was found only at 100% of MSI when comparing *Proechimys* and Wistar: 1.34 ± 0.10 and 1.72 ± 0.11 mV/ms.mV, respectively (*U* = 0, *p = 0.0153). Inset, the average of strength connection at 100% of stimulus intensity and the correspondent fEPSP. Time and amplitude of recordings are given by calibration bars on the right. *< 0.05; **≤ 0.01; ***≤ 0.001; ****≤ 0.0001.

**Figure 2:**
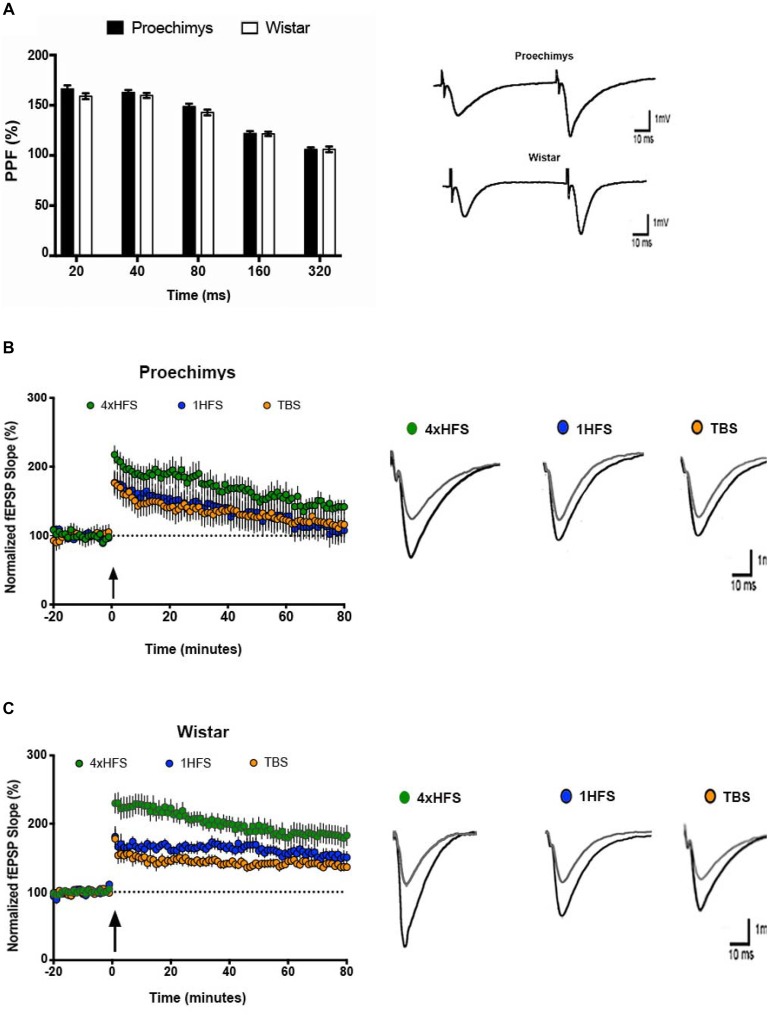
Short and long-term plasticity in *Proechimys* and Wistar rodents. **(A)** Paired pulse facilitation (PPF) at intervals of 20–320 ms. The maximum PPF was observed at an interstimulus intervals (ISIs) of 40 ms in both animal species, but neither differences nor interactions between groups were found. Insets are example or PPF recordings at 40 ms of MSI. White bars, PPF in Wistar group; black bars, PPF in *Proechimys* group at each ISI. Inset representative evoked field potentials (FPs) at interval of 40 ms. Time and amplitude of recordings are given by calibration bars on the right. **(B,C)** Long-term potentiation (LTP) was induced with three different tetanic stimulation protocols theta burst stimulation (TBS; 1HFS and 4HFS) delivered after 20 min of stable baseline recordings. **(B)** Time evolution of fEPSP slopes of *Proechimys*. Note that fEPSP slope potentiation decays through the time reaching baseline levels around 60-min after 1HFS and TBS. The gradual decay of potentiation was also observed after 4HFS, however this last stimulus elicited higher enhancement of potentiation and baseline levels were never reached during the 80-min recorded in our study. **(C)** Wistar showed enhanced and sustained potentiation with all protocols. Insets, representative superimposed recordings of averaged fEPSP before (gray trace) and 30 min after (black trace) tetanic stimulations in *Proechimys* **(B)** and Wistar **(C)**. Time and amplitude of recordings are given by calibration bars on the right.

Figure 1 shows the I/O relationships in the hippocampal CA1 area of the *Proechimys* (9/4, slices/animals) and Wistar (8/4, slices/animals) rodents. The I/O relationships were evaluated before and after the induction of LTP with 4HFS protocol. Measuring the FV amplitude and fEPSP slope at different stimulus intensity allows an evaluation of the presynaptic vs. postsynaptic response (Figures 1A,B). The FV magnitude represents the quantity of the activated presynaptic axons of the Schaffer collateral and so infers the strength of the afferent input while the fEPSP is representative of the postsynaptic depolarizing activity. Indeed, the ratio of fEPSP slope to FV amplitude at each input stimulation level enables to quantify the synaptic strength connection. When comparing this synaptic strength connection before and 80-min after 4HFS, Wistar rats (Figure 1B) presented potentiation starting at 20% of the maximal stimulus contrasting to *Proechimys* rodents (Figure 1A) that showed increased synaptic transmission only after 40% of the maximal stimulus, suggesting that stronger stimulation is required for synaptic excitatory transmission efficacy in the Neotropical rodents. As the stimulus intensity increased, in *Proechimys*, the fEPSP slope and PS amplitude were increased only after 40%–60% and 60%–80% of the maximal stimulus intensities, respectively (Figures 1C,E); however, in Wistar rats, the increases in the fEPSP slopes were observed for all intensities of stimulation whilst increases in the PS amplitude occurred only after 40%–60% of the maximal stimulus intensities (Figures 1D,F). By assuming these parameters as a measure of excitability, our data suggests lower excitability in the CA1 network of the *Proechimys*. In order to compare different animal species, a useful approach is to assess the synaptic strength connection (ratio of fEPSP slope to FV amplitude; Sweatt, 2010; Figures 1G,H). To do so, same collection of data used on Figures 1A,B were used to perform comparative analysis that produced the Figures 1G,H. The only difference between *Proechimys* and Wistar rats was observed at maximum stimulus intensity after HFS, suggesting an augmentation of synaptic transmission in Wistar rats (Figure 1H). Detailed statistical data are showed in the legend of Figure 1. The PPF was assessed in slices of both animal species. Friedman’s two-way analysis of variance with repeated measures was used on the ISIs showed significant main effects of intervals, but neither differences nor interactions between groups were found. In both animal species, the maximum PPF was observed at an interval of 40 ms, 162.7 ± 2.5% in *Proechimys* and 159.7 ± 2.4% in Wistar rats (Figure 2A).

### Long-Term Plasticity

Multiple protocols are available to induce LTP, 1HFS and TBS are commonly used. The potentiation at 30 min after 1HFS was 46.6 ± 16.3% and 67.8 ± 4.6% above baseline levels for *Proechimys* and Wistar, respectively. However, in *Proechimys* (8/4, slices/animals), fEPSP slope potentiation decayed over time reaching basal levels at 75-min after 1HFS stimulation (Figure 2B), contrasting to Wistar rats (8/4, slices/animals), in which potentiation was maintained during recordings (Figure 2C). Likewise, at 30-min after TBS, the potentiation in *Proechimys* was 34.3 ± 8.3% and in Wistar 42.7 ± 6.4%. Moreover, LTP decay was observed only in *Proechimys*. Therefore, we applied a stronger induction protocol (4HFS) in order to compare the magnitude of LTP produced in CA1 area of the hippocampal slices (4HFS vs. 1HFS/TBS; Figures 2B,C). The protocol of 4HFS induced a robust potentiation and it was chosen for all the experiments in this work. The post-tetanic potentiation after 30-min of 4HFS was 91.6 ± 21.3% and 107 ± 15.4% above baseline levels for *Proechimys* and Wistar, respectively. Our results showed that stronger stimulation was required to LTP maintenance in the wild rodents. Recordings were more stable over time in Wistar rats, however, in *Proechimys* the fEPSP slope dropped gradually even with 4HFS but LTP was maintained during the 80-min of experiment.

### Effects of D-AP5, CNQX, Bicuculline, Picrotoxin and Diazepam on LTP

**Figure 3:**
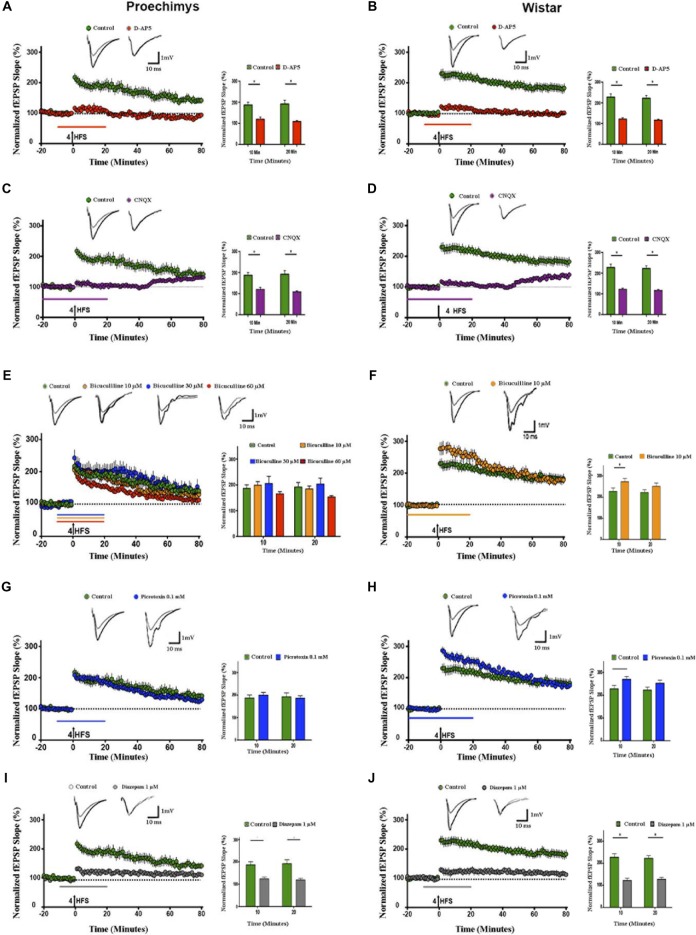
Effects of d-(-)-2-amino-5-phosphonopentanoicacid (D-AP5), CNQX, bicuculline, picrotoxin and diazepam on LTP. Slices of both species were bathed with D-AP5 (100 μM), CNQX (10 μM) bicuculline (10, 30 and 60 μM), picrotoxin (0.1 mM) and diazepam (1 μM) before and during 20 min after tetanic stimulation; see the horizontal lines insets above *x-axis* representing time periods. **(A,B)** D-AP5 and **(C,D)** CNQX prevented the LTP induction in slices of both animal species (red and purple circles, respectively). However, bicuculline (10 μM; orange circles), bicuculline (30 μM; blue circles) bicuculline (60 μM; red circles) did not enhance fEPSP slope after 4HFS in *Proechimys* **(E)**, but LTP induction was facilitated only in Wistar’s slices with bicuculline (10 μM; **F**). Recordings of control slices are represented by green circles. The graph insets represent the average of fEPSP slope at 10 and 20 min after tetanic stimulation in control slices and with bicuculline application. For Wistar, the facilitation was found only at 10 min after 4HFS (inset **F**). Superimposed recordings of average fEPSP slope: from baseline (gray trace) and after 20 min of 4HFS (black trace) for D-AP5, CNQX, control and bicuculline with different doses concentration. **(G,H)** Time evolution of fEPSP slopes of *Proechimys* and Wistar bathed with picrotoxin (0.1 mM), in *Proechimys*, fEPSP slope was not facilitated during GABA_A_ receptor antagonist **(G)**. In Wistar, fEPSP slope of slices bathed with picrotoxin is increased in comparison to control **(H)**. Inset, superimposed recordings of average fEPSP slope: baseline (gray trace) and after 20 min of 4HFS (black trace), for picrotoxin. Note that in the experiments of Wistar under GABA_A_ antagonist, the period of application of the drug was increased to allow a stable baseline before the induction of LTP. **(I,J)** Time evolution of fEPSP slopes during diazepam application. In both animal species, diazepam prevented the LTP induction (gray circles), recordings of control slices (green circles). Inset, superimposed recordings of average fEPSP slope: baseline (gray trace) and after 20 min (black trace). Graphs represent the average of fEPSP slope at the different times assessed. *< 0.05.

Since the decay of potentiation over time was observed in the slices of *Proechimys*, modulators of synaptic transmission were assessed only in LTP experiments. The D-AP5 was used to assess the effects of the NMDA-receptor mediated activity on LTP in *Proechimys* (12/5, slices/animals) and Wistar (12/5, slices/animals). Bath application of D-AP5 prevented the induction of LTP in both animal species, for example, fEPSP slope changes from baseline at 20 min after tetanic stimulation were 8.5 ± 4.6% in *Proechimys* and 12.4 ± 4.6% in Wistar (Figures 3A,B).

Then, isolated NMDA receptor was assessed using AMPA/kainate receptors antagonist (CNQX 10 μM) combined with magnesium-free aCSF. The effects of bath application with CNQX were measured in *Proechimys* (13/6 slices/animals) and Wistar (12/5 slices/animals). The potentiation of fEPSP slope were prevented in both animal species; for example, at 20 min after LTP induction, fEPSP slope changes were 6.4 ± 5.8% in *Proechimys* and 9.0 ± 5.7% in Wistar (Figures 3C,D).

Next, using bicuculline, a GABA_A_ receptor antagonist, we evaluated the effects of GABA_A_ inhibition on LTP. Interestingly, contrasting to Wistar rats (16/8, slices/animals), LTP induction was not facilitated by bicuculline in the *Proechimys* rodents (30/10, slices/animals). For example, using bicuculline, fEPSP slope changes at 10-min after tetanic stimulation in *Proechimys* were 99.1 ± 13.3 (bicuculline 10 μM, 15/10, slices/animals) and 97.8 ± 18.6% (control, 8/4, slices/animals; *U* = 46, *p* = 0.1780; Figure 3E). In Wistar rats, at 10-min after HFS: 172.9 ± 14.6% (bicuculline 10 μM, 16/8, slices/animals) and 126.0 ± 22.5% (control, 8/4, slices/animals; *U* = 42, *p = 0.0149; Figure 3F). Although the lack of fEPSP potentiation in the presence of bicuculline (10 μM) may point to a possible inhibitory network modulation of the synaptic transmission in the Neotropical rodents, another hypothesis would be that the regulatory mechanism by inhibitory network is similar in both animal species, however GABA_A_ receptors sensitivity to bicuculline is distinct, in this case GABA_A_ receptors sensitivity of *Proechimys* would be lower than that of Wistar. To test this hypothesis, experiments using bicuculline 30 μM as well as 60 μM were performed. However, no differences in fEPSP slope were encountered among all bicuculline concentrations, for example at 10-min after HFS: 99.1 ± 13.3 (bicuculline 10 μM, 15/10, slices/animals), 105.9 ± 26.6 (bicuculline 30 μM, 13/10, slices/animals), 65.3 ± 9.1% (bicuculline 60 μM, 13/10, slices/animals) and 97.8 ± 18.6% (control, 8/4, slices/animals; *H* = 3.024; *p* = 0.3880; Figure 3E). Since these findings suggested that sensitivity of the GABA_A_ receptor to bicuculline is not a crucial issue, picrotoxin (0.1 mM), a non-competitive antagonist of GABA_A_ receptors was tested on LTP induction. At 10-min after tetanic stimulation, changes in fEPSP slope in *Proechimys* (10/8, slices/animals) were 99.7 ± 12.1% and 97.8 ± 18.6% (control; *U* = 53, *p* = 0.1478; Figure 3G); in Wistar rats (16/8, slices/animals) at 10 min after HFS, fEPSP slope changes of 170.2 ± 11.9% and 126.0 ± 22.5% (control; *U* = 66, *p = 0.0067; Figure 3H). Bicuculline is a potent competitive antagonist of GABA_A_ receptors, but its binding action can be reverted by increasing the amount of substrate near to the receptor. Picrotoxin is a non-competitive antagonist of GABA_A_ receptors which binds to the allosteric site of the receptor, leading to distortion in the configuration of the active site, causing substrate inability to attach GABA to the receptor. Since neither of these two approaches were satisfactory to promote LTP potentiation, these results suggested a possible role of the GABAergic network modulation during LTP induction in the Neotropical rodents. Next, the effects of the GABA_A_ receptor positive allosteric modulator on LTP induction were assessed. Diazepam (1 μM) effectively prevented the LTP induction in slices of both animal species, for example, in *Proechimys* (10/5, slices/animals) fEPSP slope changes at 20-min after tetanic stimulation were 19.1 ± 7.7% and 97.8 ± 18.6% in control (Figure 3I; *U* = 10, **p = 0.0016); in Wistar rats (10/5, slices/animals) 27.5 ± 9.2% and 122. ± 12.9% in control (*U* = 4, ***p = 0.0007; Figure 3J).

### Effects of GABA_A_ Receptor Blockade by Bicuculline on Field Responses

**Figure 4:**
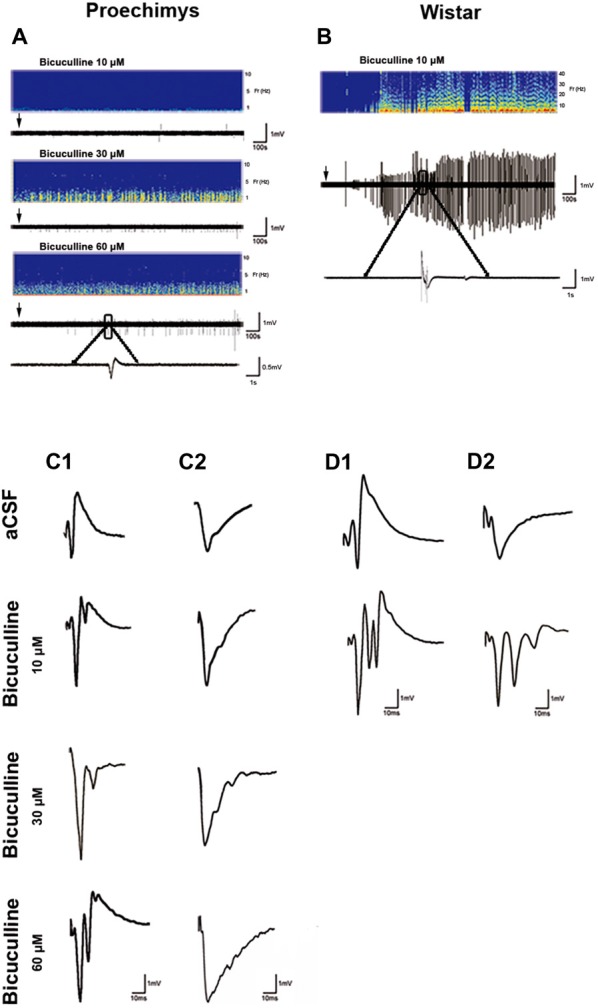
Effects of GABA_A_ receptor blockade by bicuculline on field responses. **(A,B)** show the recording of spontaneous activity after bath-applied bicuculline. Note that spontaneous activity is remarkably lower in *Proechimys*, even during increase of bicuculline concentration (10, 30 and 60 μM; **A**). For Wistar, only was assessed spontaneous activity with bicuculline at 10 μM **(B)**. Arrows indicate when bicuculline was applied. Representative spectrograms from two animals showing the corresponding burst of spontaneous activity during bicuculline bath. Panels **(C1,D1)** are samples of evoked-PS. Panels **(C2,D2)** represent evoked-fEPSP. Both were recorded in artificial cerebrospinal fluid (aCSF) and in presence of bicuculline 10 μM, 30 μM and 60 μM. Note the single recurrent PS and single fEPSP in *Proechimys*’s slices in presence of bicuculline, contrasting with the multiple bursts in Wistar recordings.

The effects of GABA_A_ inhibition on spontaneous FPs were evaluated in CA1 pyramidal cells by adding bicuculline in the perfusion bath medium in *Proechimys* (18/6, slices/animals; bicuculline 10 μM, 20 μM and 60 μM) and Wistar rats (10/3, slices/animals; bicuculline 10 μM). With 10 μM of bicuculline, slices of Wistar rats exhibited spontaneous activity in 100% of the slices (for this reason, Wistar rats were studies only with 10 μM of bicuculline). On the other hand, only 50% of *Proechimys*’s slices bathed with 10 μM of bicuculline showed spontaneous activity (*x*^2^ = 5.268, *p = 0.0220), but with 30 μM and 60 μM of bicuculline, 100% of slices showed spontaneous activity. However, number of spontaneous events were extremely different between the two species of rodents. Using 10 μM, number of events per minute was 0.38 ± 0.1 in *Proechimys* vs. 6.14 ± 0.3 in Wistar (*U* = 0.0, *p = 0.0143). In *Proechimys*, the number of events per minute was increased when analyzing all the three different concentrations (*H* = 8.138, *p = 0.0106), but the difference was observed between 10 μM and 30 μM as well as between 10 μM and 60 μM, but not between 30 μM and 60 μM (Dunn’s multiple comparison test; mean of events per minute, 10 μM = 0.38 ± 0.1; 30 μM = 1.84 ± 0.43; 60 μM = 1.81 ± 0.44). Note in Figures 4A,B the higher spontaneous activity in the Wistar rats than that of the *Proechimys*. Note the single recurrent PS and single fEPSP in *Proechimys*’s slices (Figures 4C1,C2) in presence of bicuculline, contrasting with the multiple bursts in Wistar recordings (Figures 4D1,D2).

## Discussion

Little is known about synaptic plasticity in wild Neotropical rodents. The present findings draw attention to the protocol-independent fast decay of the early-phase LTP and to the robust dynamic network inhibition assessed in the hippocampal CA1 area of the *Proechimys* rodents. The comparison between LTP expression in the presence and absence of a GABA_A_ receptor antagonist offers an indirect mensuration of alterations in inhibition and the consequent impact on excitatory responses since the inhibitory inputs and associated modifications should be inhibited by the antagonist. In Wistar rats, our results are consistent with previous findings that have shown that relief from GABA_A_ inhibition has facilitated LTP induction (Wigström and Gustafsson, 1983; Grover and Yan, 1999). In contradiction, our findings suggest that fEPSP of hippocampal CA1 pyramidal cells are not modulated by GABA_A_ inhibition during LTP induction in *Proechimys* since LTP induction was not facilitated by the presence of both bicuculline and picrotoxin.

A plausible hypothesis to explain our data would be given by differences in GABA_A_ quantification and distribution. Rocha et al. (2006) performed quantitative receptor autoradiography to assess the distribution of high-affinity GABA_A_ receptors through the powerful GABA_A_ agonist [3H] muscimol and reported no differences between *Proechimys* and Wistar rats in GABA_A_ binding quantification in the hippocampal subfields. On the other hand, it has been reported that the effect of inhibition on LTP seems to be stimulation-dependent (Chapman et al., 1998), thus the pattern of stimulation used in this study may play a role in the inhibition of population EPSP in the presence of bicuculline/picrotoxin.

Efficient synaptic transmission involves the control of brain excitability through inhibitory signaling which fundamentally occurs through GABAergic neurotransmission via ionotropic GABA_A_ receptors and the output of the adult hippocampal pyramidal neurons is strongly regulated by the activity of GABAergic cells (Bernard et al., 2000; Lee and Maguire, 2014). It seems that *Proechimys*’ brain exerts a very particular control of inhibitory modulation. For example, when 43 *Proechimys* rodents were submitted to electrical Kindling process, only three of them reached stage 5 of Racine scale and among the remaining 40 animals, 16 of them remained at stage 1, 14, 7 and 3 of them did not get beyond stages 2, 3 and 4, respectively (Racine, 1972; Arida et al., 2005). These findings suggest that *Proechimys* rodents seem incapable to consolidate an epileptic circuitry which could reflect functional alterations in inhibitory and facilitatory processes in the brain of these animals. Indeed, it has been suggested that brain stimulation (e.g., HFS) used to induce kindling are similar to LTP since both result in synaptic structural alterations and facilitation (McEachern and Shaw, 1999; Meador, 2007). Following this reasoning, previous reports described that in *Proechimys* hippocampus, parvalbumin (PV)-expressing interneurons are generally distributed and predominate in the stratum oriens, pyramidale and alveus (Fabene et al., 2001; Scorza et al., 2010). Furthermore, after an acute epileptogenic insult, Fos immediate early genes induction occurred in almost all the hippocampal PV-expressing interneurons up to 24 h after status epilepticus in *Proechimys*, contrasting to the lower proportion of the double labeled PV-Fos cells found in Wistar rats, thus suggesting differential mechanisms of GABAergic response between the two animals species (Fabene et al., 2004).

Interneurons regulate the firing rate of neurons to modulate circuitry activity. The CA1 hippocampal subfield possess a huge diversity of interneurons (Klausberger and Somogyi, 2008), thus it is possible to speculate that an inhibitory interneuron, which was innervating another inhibitory interneuron, is now inhibited in the presence of GABA_A_ antagonist and this second interneuron, that is the source of feed forward inhibition onto neurons of CA1 pyramidal layer, is now released from inhibition and hence, is able to project an inhibitory input onto CA1 pyramidal neuron. Therefore, the disinhibition implemented in the local inhibitory circuit could account for our findings in *Proechimys* but remains to be investigated.

It’s worth mentioning that LTP induction was protocol-independent in *Proechimys*, since TBS, 1HFS and 4HFS protocols reliably induced LTP but fEPSP slopes dropped gradually in all protocols, short-lived LTP was encountered after TBS/1HFS however LTP maintenance was effective after 4HFS during 80-min of recordings. As suggested by previous studies performed in guinea pigs and laboratory rats (Buzsáki and Eidelberg, 1982; Stelzer et al., 1994), it is possible that synaptic strength inhibition might be enhanced during LTP maintenance in *Proechimys*. Furthermore, the I/O relationship for fEPSP vs. FV magnitude points to lower excitability in the CA1 area of *Proechimys* (pre × post HFS). Likewise, utilizing an *in vitro* model of bicuculline (10 μM), which is broadly used to assess enhancement of electrical activity, Wistar slices exposed to bicuculline generated intense activity, in accordance with previous reports (Li et al., 2001). Notwithstanding, it is striking that, even increasing the bicuculline concentration up to 60 μM, the electrical activity was not as intense in *Proechimys* as in Wistar rats. High concentrations of bicuculline (60 microMol) decrease LTP expression at longer delay in *Proechimys* compared to control. Early-phase LTP in *Proechimys* decays very rapidly (10, 30 and 60 microMol). Previous studies reported that even weak protocols of induction can induce LTP that lasts several hours in rodents (Lu et al., 2011; Dong et al., 2015). Why early LTP in *Proechimys* decays very rapidly remains unknown. In our previous study (Guimarães Marques et al., 2018), fEPSP showed that LTP decays quickly over time reaching baseline at 90 min after TBS in *Proechimys*, in opposition to the stable LTP encountered in the Wistar rodents during 3-h period.

Previous studies in the laboratory rodents asserted that NMDA receptor activation is essential for induction of LTP (Collingridge et al., 1983; Muller et al., 1988; Murphy et al., 1997; Gruart et al., 2006). In agreement with those reports, we found the failure of LTP induction in the presence of D-AP5 and in both Wistar and *Proechimys* rodents. In the case of CNQX, HFS did not generate robust LTP. However, when the drug was removed, the potentiation effect started to appear. Therefore, NMDA receptor activation elicited LTP in both animal species, in which non-NMDA receptors were needed for the LTP expression.

This study described a rapid loss of synaptic potentiation over time and an a possibly high inhibitory tone in the CA1 network mediated by GABA_A_ receptors in the *Proechimys* rodents. Although bicuculine and picrotoxin did not modify LTP values in the *Proechimys* rodents, other aspects argue in favor of a high inhibitory tone mediated by GABA_A_ receptors, such as the lower spontaneous FPs under GABA_A_ antagonists as well the lower excitability observed in the I/O relationships evaluated before and after the induction of LTP. Nevertheless, other mechanisms could be involved such as functional saturation of the CA3-CA1 synapses through LTP induction evoked by HFS, a process that could modulate the activity-dependent synaptic plasticity and prevent acquisition of associative learning in behaving mice (Gruart et al., 2006). The inhibitory control carried by GABA_B_ receptors is another possibility to explain our findings. GABA_B_ receptors are pivotal regulators of neuronal excitability and their activation is crucial for associative learning in behaving animals (Jurado-Parras et al., 2016). Experiments assessing the roles of GABA_B_ receptor remain to be addressed in *Proechimys*. Mice lacking the GABA_B_ subunit isoform exhibited larger *in vivo* LTP than wild type (WT) mice but operant learning task impairments (Jurado-Parras et al., 2016). A contrast between increased LTP and lack of learning in operant tasks was also reported in mutant mice with deficits in GABAergic septohippocampal projections, which exhibited delayed acquisition and lower performance in the medial septum self-stimulation task, suggesting a distinctive susceptibility of the hippocampus to GABAergic inputs and cognitive processes (Vega-Flores et al., 2014). Therefore, the incorporation of robust inhibition into the hippocampal excitatory circuitry is a key ingredient in network dynamics that may crucially affect learning and memory. Previous investigation showed the poor performance of *Proechimys* rodents in plus-maze discrimination avoidance task and object recognition test since long-term memory did not last 24-h in these rodents, contrasting with the preserved 24-h memory of Wistar rats (Guimarães Marques et al., 2018). Current studies satisfy a number of the criteria needed to link learning/memory and hippocampal LTP as well as to link the conservation of long-lasting memory and LTP maintenance (Pastalkova et al., 2006; Whitlock et al., 2006; Sacktor, 2008). This study suggests a lower excitability in the hippocampus of *Proechimys* in comparison to Wistar rats. The transformation of decaying to non-decaying LTP is a process resulting from active inhibition and crucial for the transformation of short-term to long-term memory (Nguyen et al., 1994; Lu et al., 2011; Dong et al., 2015). The excitation-inhibition ratio known as E/I balance is widely accepted as a critical measure for evaluating fitness of any brain and memory preservation is costly and demands energy (Hardt et al., 2013). The brains of these rodents from Amazon rainforest may express signatures of their challenging wild ecology. At this point, a causal relation between the robust inhibitory tone in *Proechimys* and rapid early-LTP decay/poor long-term memory is unknown, and several fundamental questions arise from this investigation which remain to be addressed: (1) How the present findings effectively impact learning and memory of the *Proechimys*? (2) What are the behavioral advantages? (3) Manipulation of inhibitory network will be able to down-regulate this early-phase decay LTP in *Proechimys*? (4) How to increase the longevity of LTP and long-term memory? and (5) What are the physiological implications? Currently, neuroscience research still seeks to reveal molecular pathways that control learning and memory processes, *Proechimys* may prove useful in identifying such mechanisms in complement to traditional animal models.

## Author Contributions

All experiments were performed in the Department of Neurology/Neurosurgery, Universidade Federal de São Paulo. SR-G and NO-V together carried out the experiments and wrote the article. SR-G, A-CA, FS and EC prepared the figures and wrote the article. SR-G and CS designed the investigation and wrote the article. All persons designated as authors qualify for authorship. The authors have read and approved the final version of the article.

## Conflict of Interest Statement

The authors declare that the research was conducted in the absence of any commercial or financial relationships that could be construed as a potential conflict of interest.
